# Incomplete autophagy promotes the proliferation of *Mycoplasma hyopneumoniae* through the JNK and Akt pathways in porcine alveolar macrophages

**DOI:** 10.1186/s13567-022-01074-5

**Published:** 2022-08-04

**Authors:** Yukang Wen, Zhengkun Chen, Yaqin Tian, Mei Yang, Qingshuang Dong, Yujiao Yang, Honglei Ding

**Affiliations:** Laboratory of Veterinary Mycoplasmology, College of Veterinary Medicine, Southwest University, Chongqing, 400715 China

**Keywords:** LC3, porcine alveolar macrophage, immunofluorescence assay, pig, autophagosome

## Abstract

Autophagy is an important conserved homeostatic process related to nutrient and energy deficiency and organelle damage in diverse eukaryotic cells and has been reported to play an important role in cellular responses to pathogens and bacterial replication. The respiratory bacterium *Mycoplasma hyopneumoniae* has been identified to enter porcine alveolar macrophages, which are considered important immune cells. However, little is known about the role of autophagy in the pathogenesis of *M. hyopneumoniae* infection of porcine alveolar macrophages. Our experiments demonstrated that *M. hyopneumoniae* infection enhanced the formation of autophagosomes in porcine alveolar macrophages but prevented the fusion of autophagosomes with lysosomes, thereby blocking autophagic flux and preventing the acidification and destruction of *M. hyopneumoniae* in low-pH surroundings. In addition, using different autophagy regulators to intervene in the autophagy process, we found that incomplete autophagy promoted the intracellular proliferation of *M. hyopneumoniae*. We also found that blocking the phosphorylation of JNK and Akt downregulated the autophagy induced by *M. hyopneumoniae*, but pathways related to two mitogen-activated protein kinases (Erk1/2 and p38) did not affect the process. Collectively, *M. hyopneumoniae* induced incomplete autophagy in porcine alveolar macrophages through the JNK and Akt signalling pathways; conversely, incomplete autophagy prevented *M. hyopneumoniae* from entering and degrading lysosomes to realize the proliferation of *M. hyopneumoniae* in porcine alveolar macrophages. These findings raise the possibility that targeting the autophagic pathway may be effective for the prevention or treatment of *M. hyopneumoniae* infection.

**Supplementary Information:**

The online version contains supplementary material available at 10.1186/s13567-022-01074-5.

## Introduction

*Mycoplasma hyopneumoniae* is a serious threat to global pork production and is one of the smallest and simplest self-replicating bacteria. This pathogen lacks a cell wall and has a genome size of approximately 900 kb [1]. It is considered the aetiological agent of porcine enzootic pneumonia (EP) and has a broad geographic distribution [2]. Under conditions of modern intensive breeding, pigs infected with *M. hyopneumoniae* typically do not show overt signs of infection, and EP generally does not cause mortality. However, persistent infection caused by this pathogen can lead to reduced feed conversion and production performance [3]. The pathogenic mechanisms of persistent infection by *M. hyopneumoniae* are not well characterized.

Autophagy is an important homeostatic process in eukaryotic cells that can be triggered by nutrient deficiency, endoplasmic reticulum stress, and pathogen infection. The hallmark of autophagy is the formation of double-membrane vesicles—autophagosomes—that sequester and transport damaged cytoplasmic components or organelles and invading intracellular pathogens into lysosomes for degradation [4]. Generally, the formation of autophagosomes is an intermediate step in the process of autophagy; after their formation, autophagosomes fuse with lysosomes to form autolysosomes, followed by enzymatic degradation of the contents. Thus, the aggregation of autophagosomes may reflect the production or nondegradation of autophagosomes. Recent studies have demonstrated the important role of autophagy in the pathogenesis of bacterial infection. For example, *Streptococcus suis* serotype 2 (SS2) infection was shown to activate microglial autophagy and enhance autophagic flux, leading to a host defence response against SS2 in microglial cells [5]. In addition, the *Acinetobacter baumannii* OmpA protein induces autophagy but disrupts the fusion of autophagosomes with lysosomes by altering the mitogen-activated protein kinases/c-Jun NH2-terminal kinase (MAPK/JNK) signalling pathway [6].

After entering the porcine upper respiratory tract, *M. hyopneumoniae* interacts with cilia on the epithelial surface lining the trachea, bronchi, and bronchioles. Colonization of the upper respiratory tract by *M. hyopneumoniae* results in the destruction of the mucociliary escalator due to ciliostasis, loss of cilia, and eventual epithelial cell death [7]. Next, *M. hyopneumoniae* invades the lungs, causing well-demarcated, dark purple, atelectatic pathology in the anterior cranial and middle lung lobes and the apical anterior portion of the caudal lobe of the lungs [8]. A recent study demonstrated the colonization of porcine type I and type II alveolar cells and porcine alveolar macrophages (PAMs) by *M. hyopneumoniae* [8, 9]. In our previous studies, *M. hyopneumoniae* infection was shown to increase the number of autophagosomes and bacterial replication in lung cells [10]. Another in vitro study reported increased generation of autophagosomes in a porcine alveolar macrophage cell line 3D4/21 that was infected with *M. hyopneumoniae* [11]. Macrophages can phagocytose and kill pathogens in cells and can also function as antigen-presenting cells. Studies have indicated that macrophages process pathogens into antigen peptides, which bind to major histocompatibility complex (MHC) I/II molecules to form antigen peptide-MHC I/II molecular complexes that are presented to CD4^+^ and CD8^+^ T cells [12]. These findings led to our hypothesis that *M. hyopneumoniae* induces autophagy in PAMs, followed by a series of pathological cellular events, including interfering with the process of autophagy and the proliferation of *M. hyopneumoniae*.

In the present study, we used freshly harvested PAMs and cultured 3D4/21 cells to investigate the relationship between autophagy and *M. hyopneumoniae* infection and to clarify the effect of autophagy on the intracellular proliferation of *M. hyopneumoniae*. We also examined the potential signalling pathways of *M. hyopneumoniae*-induced autophagy.

## Materials and methods

### Ethics statement

The protocol for the collection of PAMs from pigs after their slaughter was approved by the Institutional Animal Care and Use Committee of Southwest University. All experiments were carried out in accordance with the Guidelines for the Care and Use of Laboratory Animals of the Ministry of Health of China. Written informed consent was obtained from the owner of the pigs.

### Reagents and antibodies

An LC3B polyclonal antibody (GTX127375) was purchased from GeneTex (Irvine, CA, USA). Polyclonal antibodies against ATG5 (NBP2-24389), ATG5 (NB110-53818), Beclin 1 (NB110-87318), and p62 (NBP1-48320) were purchased from Novus Biologicals (Shanghai, China). HRP-conjugated goat anti-rabbit IgG (BL003A) was obtained from Biosharp (Hefei, Anhui, China). HRP-conjugated goat anti-mouse IgG (SE131) and the CCK-8 cell proliferation and cytotoxicity assay kit (CA1210) were acquired from Solarbio (Beijing, China). Primary antibodies against β-actin (66009-1-Ig), p38 MAPK (66234-4-Ig), lysosome-associated membrane protein 2 (LAMP2) (66301-1), and extracellular signal-regulated kinases 1 and 2 (Erk1/2) (16443-1-AP) and fluorescent secondary antibodies (CoraLite594-conjugated donkey anti-rabbit IgG [SA00006-8] and Alexa Fluor 488-conjugated AffiniPure goat anti-mouse IgG [SA00006-1]) were obtained from Proteintech (Wuhan, China). Primary antibodies against JNK (bs-2592R), p-JNK (bs-1640R), p-Erk1/2 (bs-3016R), p-p38 MAPK (bs-2210R), protein kinase B (Akt) (bs-0115R), and p-Akt (bs-5182R) and an HRP-conjugated goat anti-mouse IgG secondary antibody (bs-0296G-HRP) were purchased from Biosynthesis Biotechnology (Beijing, China). The Erk1/2 inhibitor PD98059 (S1805), JNK inhibitor SP600125 (S1876), Akt inhibitor LY294002 (S1737), and Hoechst 33342 staining solution for live cells (C1028) were purchased from Beyotime Biotechnology (Beijing, China). The adenovirus Ad-mRFP-GFP-LC3 (HB-AP2100001) was purchased from Hanbio (Shanghai, China). The autophagy activator rapamycin (Rap, V900930) was purchased from Sigma–Aldrich (Shanghai, China). The autophagy inhibitors hydroxychlorotuine sulfate (HCQ, S4430) and 3-methyladenine (3-MA, S2767) were obtained from Selleck (Shanghai, China), and bafilomycin A1 (BAF, A8627) was obtained from APExBIO (Shanghai, China). The monoclonal antibody against P97 was provided by Dr Guoqing Shao and Dr Zhixin Feng (Institute of Veterinary Medicine, Jiangsu Academy of Agricultural Sciences). Mhp366 polyclonal antibody was previously prepared by our lab [9].

### Porcine alveolar macrophages

Two types of whole-lung specimens (including the trachea) of pigs, regardless of sex, were collected immediately after slaughter at a slaughterhouse in Chongqing, China. These pigs were sourced from 4 pig farms in Chongqing. Some pigs in these farms showed clinical signs of EP and exhibited seropositivity for *M. hyopneumoniae*. One type of lung specimen appeared to be devoid of any lesions and was pink in colour, and the other type of lung had bilateral (generally) cranioventral pulmonary consolidation (CVPC) in the apical, intermediate, accessory, and cranial portions of the diaphragmatic lobes. Each lung and tracheal specimen was lavaged with 100 mL of sterilized phosphate buffered saline (PBS) containing 100 μg/mL ampicillin and 2 μg/mL kanamycin. Each lung was then rubbed gently to promote full contact of the PBS with the alveoli for 3 min and was then placed upside down to collect the bronchoalveolar lavage fluid (BALF). This procedure was performed twice. The BALF was then passed through 3 layers of sterile gauze and centrifuged at 200 × *g* for 10 min at 4 ℃. The supernatant was discarded, and the pellet was resuspended in PBS that contained 100 μg/mL ampicillin and 2 μg/mL kanamycin. Infection by *M. hyopneumoniae* was confirmed by using nested polymerase chain reaction (PCR) for detection of the *P36* gene [13]. Samples were then used for culture, transmission electron microscopy (TEM), or Western blotting.

### M. hyopneumoniae strain and cell culture

The AH strain of *M. hyopneumoniae* (henceforth, AH), which was provided by Dr Guoqing Shao, was cultured in KM2 medium (prepared by the addition of 5 g lactalbumin hydrolysate, 10 g fresh yeast extract, 5.975 g Eagle’s solution, 2.925 g Dulbecco’s phosphate-buffered saline, and 0.007 g phenol red into 1 L ddH_2_O) containing 20% (v/v) swine serum [14]. When the cells had reached the logarithmic growth stage, the cell density was calibrated to 1 × 10^8^ colour changing units/mL (CCU/mL). The collected PAMs were counted and seeded into 24-well plates at a density of 3 × 10^5^ cells/well with RPMI 1640 medium supplemented with 10% FBS, 100 μg/mL ampicillin and 2 μg/mL kanamycin to prevent contamination by other bacteria. Cultured 3D4/21 cells (ATCC, CRL-2843) were seeded into 6- or 24-well plates and cultured in DMEM (containing 4500 mg/L D-glucose, 110 mg/L sodium pyruvate, 584 mg/L L-glutamine, 200 mg/L CaCl_2_, and 97.67 mg/L MgSO_4_) with 10% FBS or starved in EBSS (containing 1000 mg/L D-glucose, 200 mg/L CaCl_2_, 2200 mg/L NaHCO_3_, and 200 mg/L MgSO_4_·H_2_O). All cells were maintained in a humidified incubator with 5% CO_2_ at 37 ℃.

### Optimal concentrations of autophagy regulators and cytotoxicity assay

The 3D4/21 cells in 24-well plates were allowed to achieve 60% confluence. After washing twice with PBS, cells were incubated for 6 h in different concentrations of 3-MA (to block the initiation of autophagy and the maturation of autophagosomes), rapamycin (autophagy inducer; to induce the production of autophagosomes), HCQ (to cause an increase in lysosomal/vacuolar pH, and, ultimately, block the fusion of autophagosomes with vacuoles), or BAF (has the same function as HCQ) before harvest. Before use, 3-MA was dissolved in DMEM + 10% FBS at a concentration of 100 mmol/L, and rapamycin, HCQ, and BAF were dissolved in dimethyl sulfoxide (DMSO) to 100 μmol/L, 5 mmol/L, and 10 μmol/L as stock solutions. These cells were collected, and Western blotting was performed to determine the levels of LC3-II and p62. The optimal working concentration of each drug (i.e., the lowest concentration at which the response was maximal) was used for subsequent experiments.

The cytotoxicities of 3-MA, rapamycin, HCQ, and BAF were assessed using the optimal working concentration of each drug. Briefly, 3D4/21 cells were seeded into 96-well plates at a density of 1 × 10^3^ cells/well and cultured overnight in a 5% CO_2_ cell incubator at 37 °C. After washing with PBS twice, the optimal working concentration of each agent was dissolved in 0.1 mL of DMEM + 10% FBS and added to each well, followed by incubation for 6 h. Next, 10 μL of CCK-8 medium was added to each well without discarding the culture medium, and the plates were then cultured for another 2 h at 37 ℃. The optical density at 450 nm was measured using an xMark™ microplate spectrophotometer (Bio-Rad).

### Drug treatments

In our previous study, viability of 3D4/21 cells infected with the AH strain was the highest at a multiplicity of infection (MOI) of 200 [9]. To measure autophagic flux, after the 3D4/21 cells in 24- or 6-well plates had attained 60% confluence, the cells were infected with AH at an MOI of 200 for 3 h. After washing twice with PBS, the optimal working concentration of HCQ or BAF was added to each well, and the cells were cultured for 3 or 6 h. The experimental flow is shown in Additional file 1A.

For analysis of signalling pathways, when the 3D4/21 cells in 24- or 6-well plates had attained 60% confluence, an Erk1/2 inhibitor (PD98059, 10 mmol/L), JNK inhibitor (SP600125, 10 mmol/L), or Akt inhibitor (LY294002, 10 mmol/L), each dissolved in DMEM + 10% FBS, was added for 1 h. Then, cells were infected with AH at an MOI of 200 and treated with autophagy inhibitor for 9 h until the samples were harvested. The experimental flow is shown in Additional file 1B.

For quantification of intracellular *M. hyopneumoniae*, infected cells were treated with different regulators of autophagy. For 3-MA experiments, 3D4/21 cells were grown to 60% confluence in 6-well plates, pretreated with 3-MA for 6 h, infected with AH (MOI: 2 or 200) and then treated with 3-MA again for 7 h. For rapamycin, HCQ, and BAF experiments, AH (MOI: 2 or 200) was used to infect 3D4/21 cells after they had achieved 60% confluence; after 3 h, the cells were washed twice with PBS to remove unattached bacteria and were then incubated in culture medium supplemented with rapamycin, HCQ, or BAF for 4 h. After treatment with different autophagy regulators, cells were washed twice with PBS to remove the drugs and then incubated with 100 µg/mL gentamicin for 2 h. Cells were harvested by adding cell lysis buffer (containing 1% Triton X-100, 20 mmol/L Tris (pH 7.5), 150 mmol/L NaCl, 1 mmol/L PMSF, and some inhibitors, such as sodium pyrophosphate, β-glycerophosphate, EDTA, Na_3_VO_4_, and leupeptin) and incubated in an ice water bath for Western blotting or were treated with trypsin and ultrasonication for bacterial culture and CCU counting in KM2 medium. The experimental flow is shown in Additional file 1C.

### Transmission electron microscopy

PAMs and 3D4/21 cells detached from 24-well plates with trypsin and washed 3 times with PBS, with or without *M. hyopneumoniae* infection, were resuspended with 0.5% glutaraldehyde and then centrifuged at 200 × *g* for 10 min at 4 ℃. The supernatant was discarded, and the pellets were incubated with 3% glutaraldehyde for 1 h at room temperature (RT) and then fixed with 1% osmium tetroxide. The cells were dehydrated by passage through a graded acetone series (30%, 50%, 70%, 80%, 90%, 95%, 100% and 100%). Ultrathin sections (approximately 50 nm) were prepared by using a microtome to cut agglutinated cells that were embedded in an epoxy resin. Autophagosome-like vesicles were observed using transmission electron microscopy (TEM, JEM-1400PLUS, JEOL Ltd.) after staining with uranium acetate and lead citrate.

### Indirect immunofluorescence assay

After infection or treatment, the cells were washed 3 times with PBS and then fixed with 4% paraformaldehyde for 1 h at RT. Cell monolayers were permeabilized with 0.2% Triton X-100 for 15 min, blocked with 5% BSA for 1 h, and then incubated with specific primary antibodies and secondary antibodies conjugated with CoraLite 594 or Alexa Fluor 488 fluorophore. Nuclei were stained with DAPI at RT for 5 min in the dark. The adenovirus Ad-mRFP-GFP-LC3 was used to infect 3D4/21 cells to trace autophagic flux. Briefly, 0.5 mL DMEM + 10% FBS containing 1 × 10^5^ 3D4/21 cells was added to each well of a 24-well plate and cultured in a 5% CO_2_ cell incubator at 37 ℃ until 40% confluence was reached. Then, 0.25 mL of adenovirus Ad-mRFP-GFP-LC3 dissolved in DMEM + 10% FBS at an MOI of 20 was added to each well to infect 3D4/21 cells. After culture at 37 ℃ for 2 h, 0.25 mL DMEM + 10% FBS was added to each well. After 6 h of infection, the liquid was discarded, and the cells were infected with the AH strain at an MOI of 200 for 9 h and then treated as described above. Fluorescence was examined using fluorescence microscopy (Olympus IX71, Tokyo, Japan).

### Western blotting

PAMs were washed twice with cold PBS containing 100 μg/mL ampicillin and 2 μg/mL kanamycin, and 3D4/21 cells were washed with cold PBS three times. After that, the cells were lysed on ice for 30 min in cell lysis buffer. Then, the lysates were centrifuged at 4 ℃ for 5 min at 8600 × *g* to remove cell debris, and the protein concentration was determined by a BCA protein assay kit. Equal amounts of protein samples were boiled at 105 ℃ for 10 min in 2 × SDS–PAGE loading buffer. Proteins were resolved in SDS–PAGE gels (12–15%) and then transferred onto polyvinylidene fluoride (PVDF) membranes. After blocking in 5% skim milk dissolved in TBST overnight at 4 ℃ or at RT for 2 h, the membranes were incubated overnight with the primary antibody at 4 ℃ and then with the secondary antibody conjugated to horseradish peroxidase (HRP) at RT for 1 h at the appropriate dilution. The antigen–antibody complex was visualized by using an electrochemiluminescence (ECL) kit. The protein bands were obtained from a Molecular Imager® ChemiDoc™ XRS + imaging system and quantified by densitometry using ImageJ version 1.42 (National Institutes of Health, USA).

### Statistical analysis

All experiments were performed at least three times. Clinical samples of each type were collected from no less than 10 pigs. The numbers of autophagic vacuoles and fluorescent puncta were counted from no less than 5 cells, and the data are expressed as the mean ± standard deviation (SD). Between-group differences were assessed using a *t* test. Multigroup comparisons were performed using one-way ANOVA. Data were plotted using GraphPad Prism (version 8.0). The level of significance was indicated as ^*^ (*p* ≤ 0.05) or ^**^ (*p* ≤ 0.01), and *p* > 0.05 was considered statistically nonsignificant.

## Results

### ***M. hyopneumoniae*** infection induces autophagy in porcine alveolar macrophages

**Figure 1:**
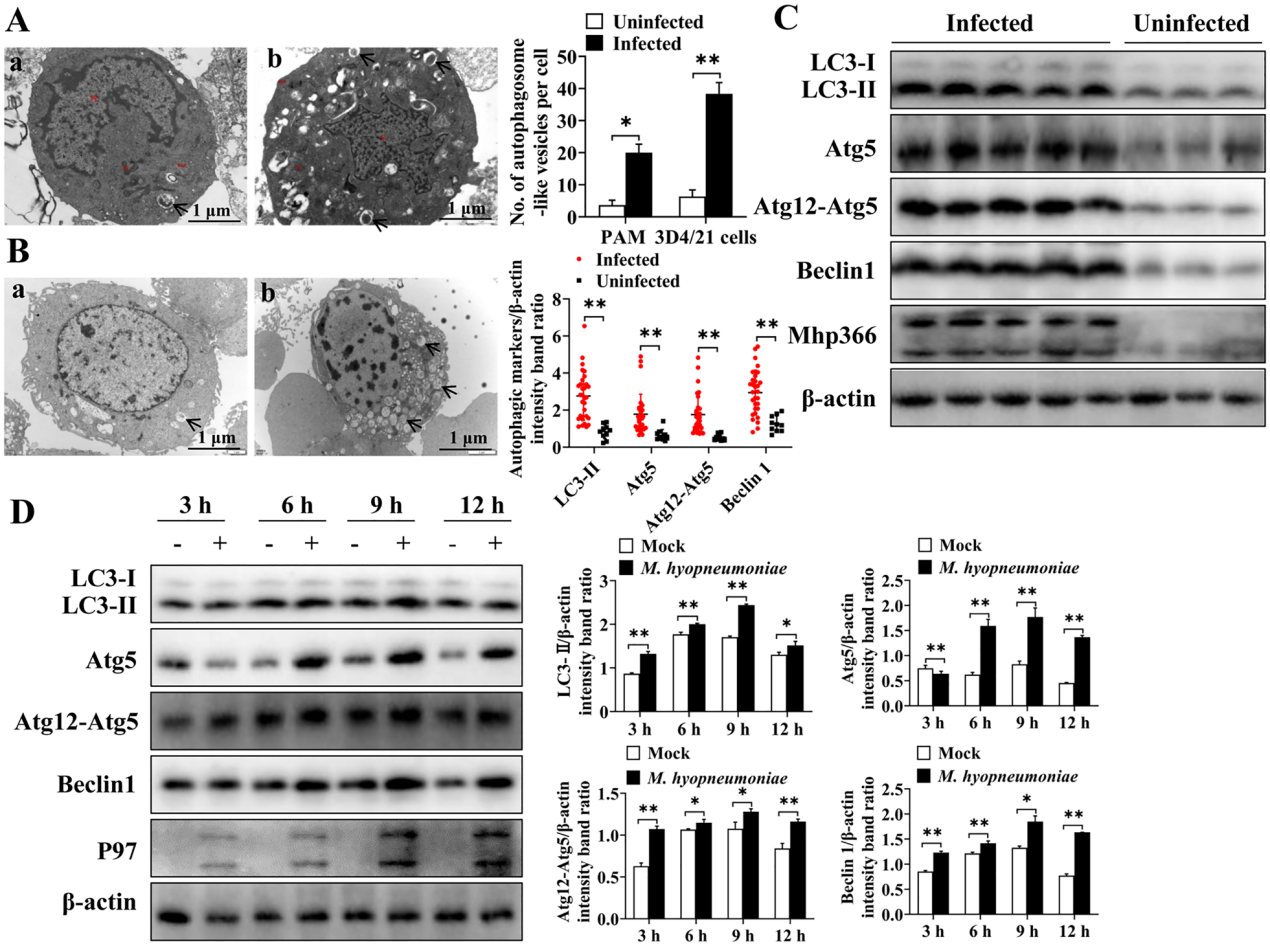
***M. hyopneumoniae*** **infection increases the formation of autophagosome-like vesicles and the expression of autophagy-related proteins in porcine alveolar macrophages**. **A** Autophagosome-like vesicles (arrows) were observed in *M. hyopneumoniae*-negative (a) and *M. hyopneumoniae*-positive (b) PAMs by TEM. Ten *M. hyopneumoniae*-negative and 10 M*. hyopneumoniae*-positive PAMs were assessed. **B** Autophagosome-like vesicles (arrows) were observed in mock-infected (a) or AH-infected (b) 3D4/21 cells by TEM. Ten uninfected and 10 AH-infected 3D4/21 cells were assessed. **C** Results of Western blotting showing the expression of autophagy-related proteins (LC3-II, Atg5, Atg12-Atg5, and Beclin 1) and Mhp366 in 30 M*. hyopneumoniae*-positive and 10 M*. hyopneumoniae*-negative PAMs collected from pigs. **D** Results of Western blotting showing the expression of autophagy-related proteins (LC3-II, Atg5, Atg12-Atg5, and Beclin 1) and P97 protein in 3D4/21 cells after mock infection or AH infection. Mean values from 3 independent experiments are presented. Between-group differences were assessed using a *t* test; *p ≤ 0.05, **p ≤ 0.01.

To illuminate whether *M. hyopneumoniae* could induce autophagy in PAMs, several experiments were performed. TEM is considered the gold standard method for identifying the ultrastructure of autophagic compartments and observing the formation of double-membrane autophagic vacuoles. Thus, we detected the formation of autophagosomes in *M. hyopneumoniae*-infected cells by TEM and compared the infected cells with *M. hyopneumoniae-*negative PAMs and mock-infected 3D4/21 cells. We first observed the number of autophagosomes in *M. hyopneumoniae*-positive and *M. hyopneumoniae*-negative PAMs. As demonstrated in Figure 1A, the number of autophagosomes was significantly greater in *M. hyopneumoniae*-positive cells (*p* ≤ 0.05). Because the presence of other pathogens in the isolated PAMs was not detected, we could not determine whether the increased autophagic vacuoles were caused by the infection of *M. hyopneumoniae* or by the invasion of other pathogens. Thus, to confirm that *M. hyopneumoniae* triggered PAMs to produce the “onion-like” structure of autophagosomes, we calculated the number of autophagosome-like vesicles in AH-infected and mock-infected 3D4/21 cells. The results indicated that the number of autophagic vacuoles in 3D4/21 cells infected with the AH strain was significantly greater than that in the uninfected group (*p* ≤ 0.01) (Figure 1B).

To further determine whether *M. hyopneumoniae* could induce autophagy in PAMs, a Western blotting assay was performed to measure the expression of autophagy-related proteins. A total of 40 PAM samples were collected in this study, including 10 from pigs with *M. hyopneumoniae*-negative cells and no gross lesions and 30 from pigs with *M. hyopneumoniae*-positive cells and EP-like gross lesions. The results indicated that the expression levels of LC3-II (*p* ≤ 0.01), Atg5 (*p* ≤ 0.01), Atg12-Atg5 (*p* ≤ 0.01), and Beclin 1 (*p* ≤ 0.01) in *M. hyopneumoniae*-positive cells were significantly greater than those in *M. hyopneumoniae*-negative cells (Figure 1C). This result was also verified in a cell line. The expression levels of LC3-II, Atg5, Atg12-Atg5, and Beclin 1 in AH strain-infected 3D4/21 cells collected at different time points (3 h, 6 h, 9 h and 12 h) were significantly greater than those in the uninfected group (Figure 1D). Moreover, the expression levels of autophagy-related proteins gradually increased with time, reaching peak levels at 9 h.

**Figure 2:**
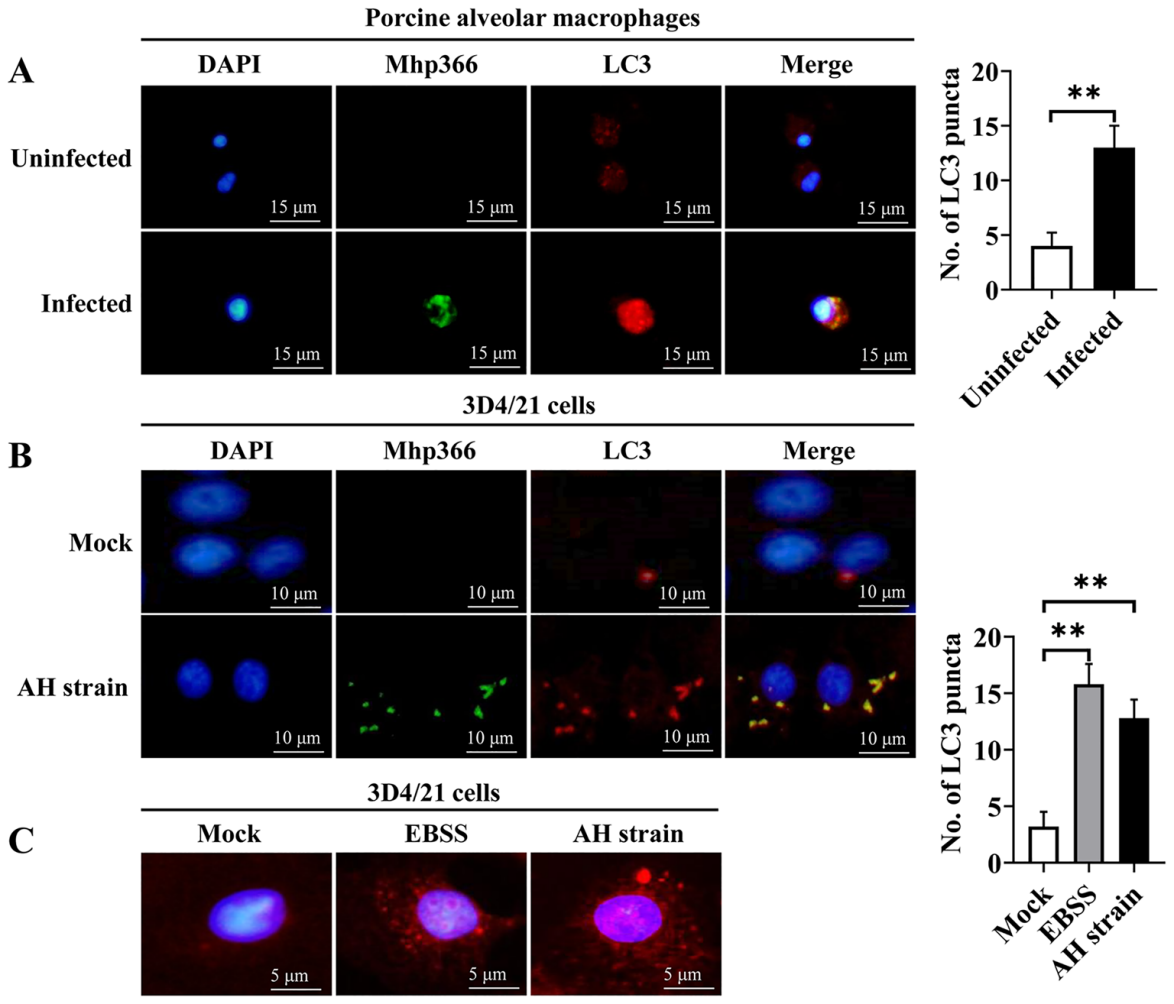
**Immunofluorescence analysis of the accumulation of** ***M. hyopneumoniae*** **and colocalization with LC3 after infection**. **A** Colocalization of Mhp366 (green) and LC3 (red) in *M. hyopneumoniae*-positive and *M. hyopneumoniae*-negative PAMs. **B** Colocalization of Mhp366 (green) and LC3 (red) in 3D4/21 cells at 9 h after infection by AH. **C** Number of LC3 dots in mock-infected, EBSS-starved and AH-infected 3D4/21 cells. Mean ± SD values from 5 independent experiments were compared using a *t* test (**A**) or one-way ANOVA (**C**); **p ≤ 0.01.

Other reliable markers of autophagy activation are colocalization of the pathogen and LC3 and the punctate accumulation of LC3. Thus, we cultured freshly collected PAMs in 24-well plates and performed immunofluorescence staining to assess the colocalization of Mhp366 (a marker of *M. hyopneumoniae*) and LC3. The results indicated that only red fluorescence (LC3) was detected in *M. hyopneumoniae*-negative PAMs; however, yellow fluorescent puncta, due to the colocalization of Mhp366 (green) and LC3 (red fluorescent dots), were observed in *M. hyopneumoniae*-positive PAMs (Figure 2A). The number of LC3 fluorescent signals in *M. hyopneumoniae*-positive cells was significantly greater than that in *M. hyopneumoniae*-negative cells (*p* ≤ 0.01). A similar phenomenon was observed in 3D4/21 cells 9 h after infection with the AH strain, i.e., the colocalization of Mhp366 and LC3 was observed (Figure 2B); in addition, the number of LC3 fluorescent dots in the infected group, similar to autophagic stimuli by starvation (EBSS group), was much higher than that in the uninfected group (*p* ≤ 0.01) (Figure 2C). All the above results indicated that *M. hyopneumoniae* infection induces autophagy in PAMs.

### Interference of autophagosome and lysosome fusion by *M. hyopneumoniae* decreases autophagic flux

Several drugs were used in this study to inhibit the initiation of autophagy (3-MA), induce the production of autophagosomes (rapamycin), or block the fusion of autophagosomes and lysosomes (HCQ and BAF). First, we performed experiments to determine the optimal working concentration of each regulator. As shown in Additional file 2, the optimal working concentrations of 3-MA, rapamycin, HCQ, and BAF were 5 mmol/L, 1000 nmol/L, 25 µmol/L, and 200 nmol/L, respectively. Importantly, none of these drugs had cytotoxic effects on 3D4/21 cells at these concentrations (Additional file 3).

**Figure 3:**
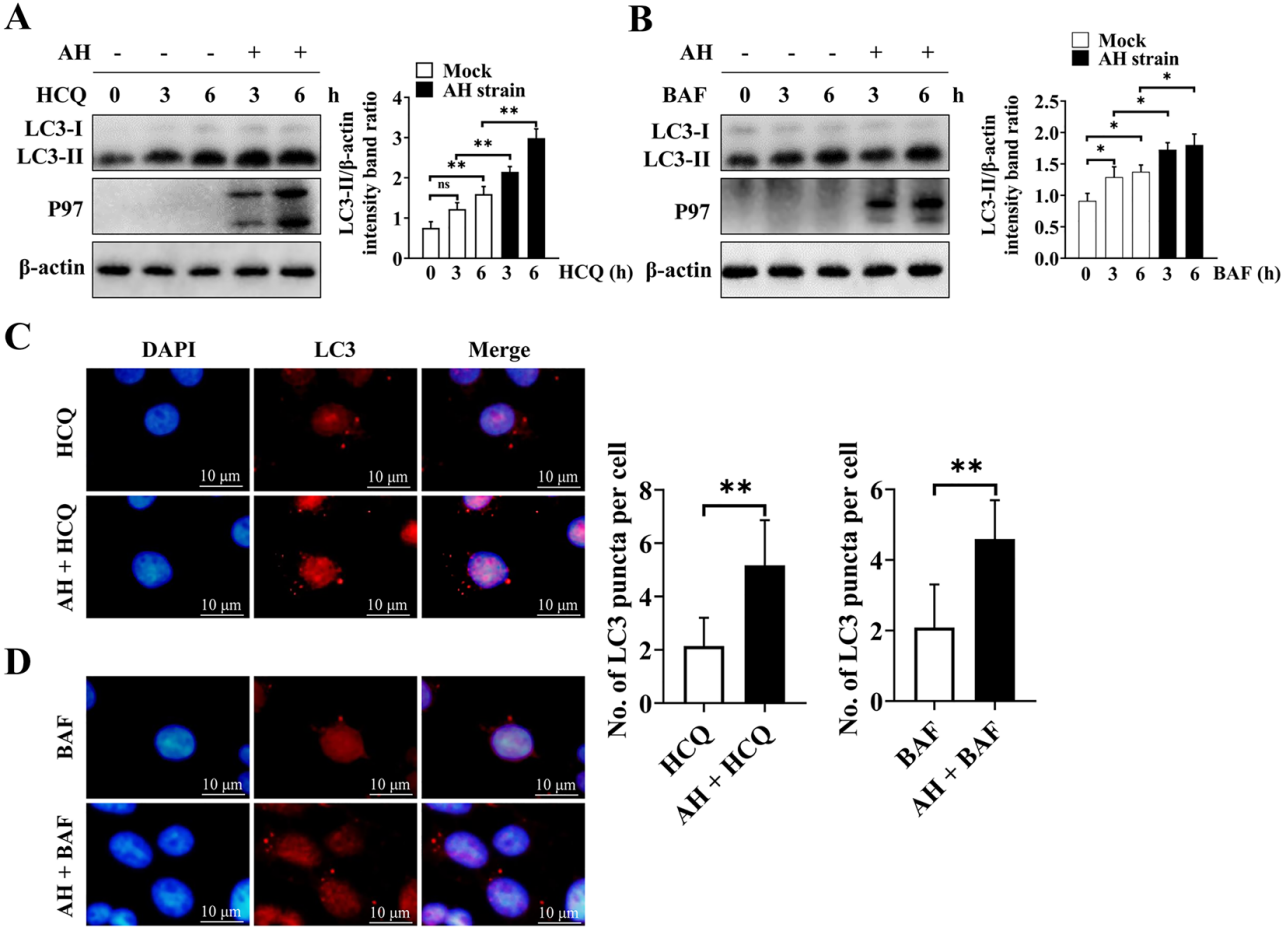
**Accumulation of LC3 after treatment with different autophagy inhibitors in 3D4/21 cells with or without infection by the AH strain**. **A** Western blotting of LC3-II in AH-infected cells and mock-infected cells after treatment with HCQ for different durations. **B** Western blotting of LC3-II in AH-infected cells and mock-infected cells after treatment with BAF for different durations. **C** Immunofluorescence of LC3 puncta (red) in AH-infected and uninfected 3D4/21 cells that were treated with HCQ for 6 h. **D** Immunofluorescence of LC3 puncta (red) in AH-infected and uninfected 3D4/21 cells that were treated with BAF for 6 h. Mean values from 3 independent experiments are presented in **A** and **B**; 35 cells from each treatment were counted to generate the data shown in **C** and **D**. Statistical significance was determined using one-way ANOVA (**A**, **B**) or a *t* test (**C**, **D**). ns: not significant, *p ≤ 0.05, **p ≤ 0.01.

**Figure 4:**
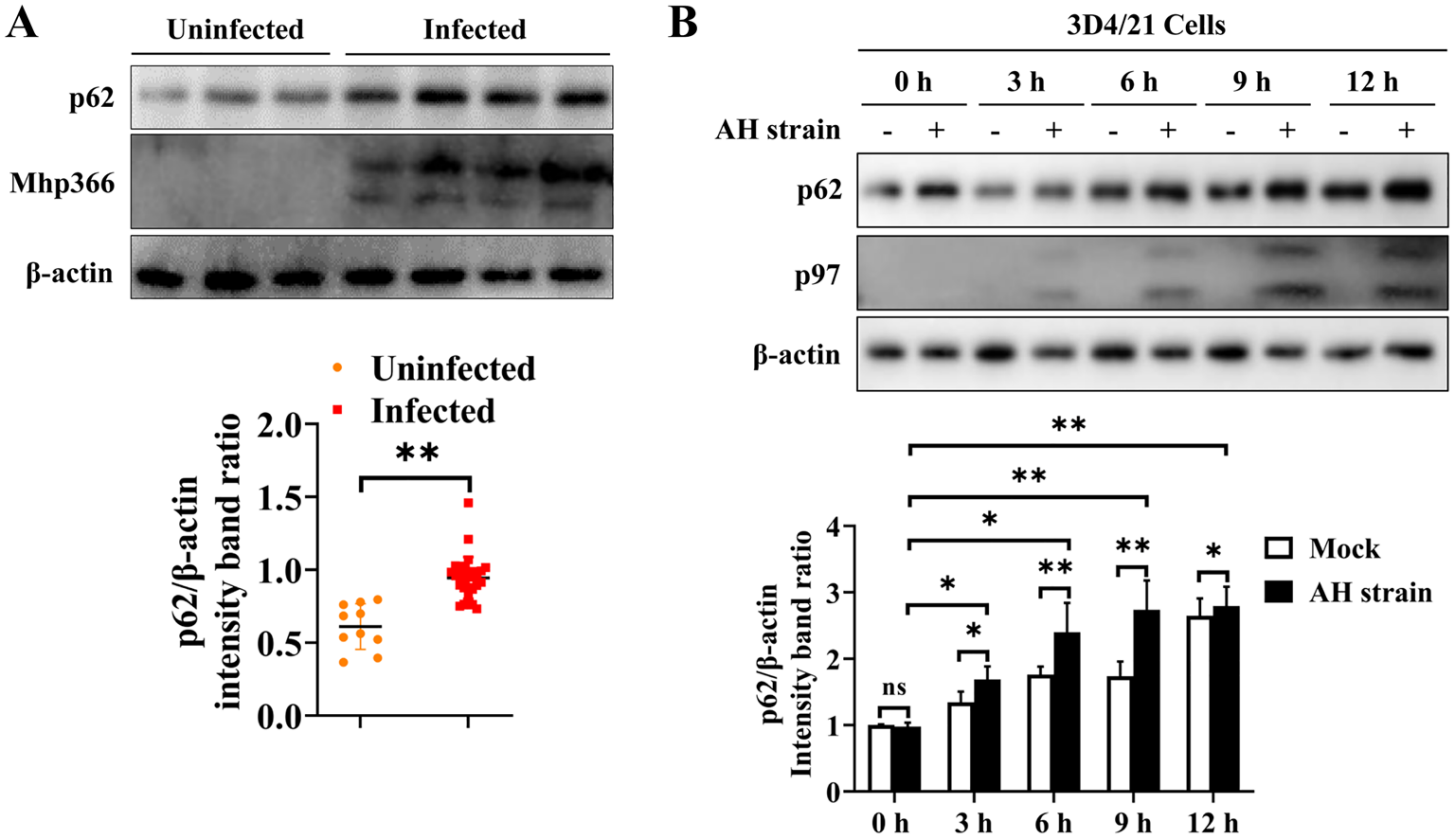
**Expression of p62 protein in freshly collected PAMs or 3D4/21 cells with or without infection by** ***M. hyopneumoniae***. **A** Western blotting of p62 in PAMs infected with *M. hyopneumoniae* and uninfected PAMs. The results are presented from 10 M*. hyopneumoniae*-negative and 30 M*. hyopneumoniae*-infected PAMs. Between-group differences were assessed using a *t* test. **B** Western blotting of p62 in AH-infected and uninfected 3D4/21 cells over time. Mean ± SD values from 3 independent experiments were compared using a *t* test or one-way ANOVA. ns: not significant, *p ≤ 0.05, **p ≤ 0.01.

Autophagosomes are merely the intermediate products within the autophagic flux. The accumulation of autophagosomes is the result of the increased generation of autophagosomes or impaired autophagosome-lysosome fusion. As shown in Figure 3A, a high level of LC3-II expression was observed in AH strain-infected 3D4/21 cells compared to mock-infected cells following HCQ treatment. The same phenomenon was observed in the BAF-treated groups (Figure 3B). Verification of these results using immunofluorescence showed that the number of LC3 fluorescent puncta in AH-infected 3D4/21 cells that were treated with HCQ was significantly greater than that in uninfected cells (*p* ≤ 0.01) (Figure 3C). The same result was obtained from groups treated with BAF (*p* ≤ 0.01) (Figure 3D). We further evaluated the expression of p62, which binds to LC3; these two proteins are degraded in the completed autophagy process after the fusion of autophagosomes with lysosomes. The expression of p62 in freshly collected *M. hyopneumoniae*-infected PAMs was found to be significantly higher than that in PAMs without *M. hyopneumoniae* infection (Figure 4A). A similar result was observed in AH-infected 3D4/21 cells, and the peak expression of p62 was observed at 9 h after infection (Figure 4B).

**Figure 5:**
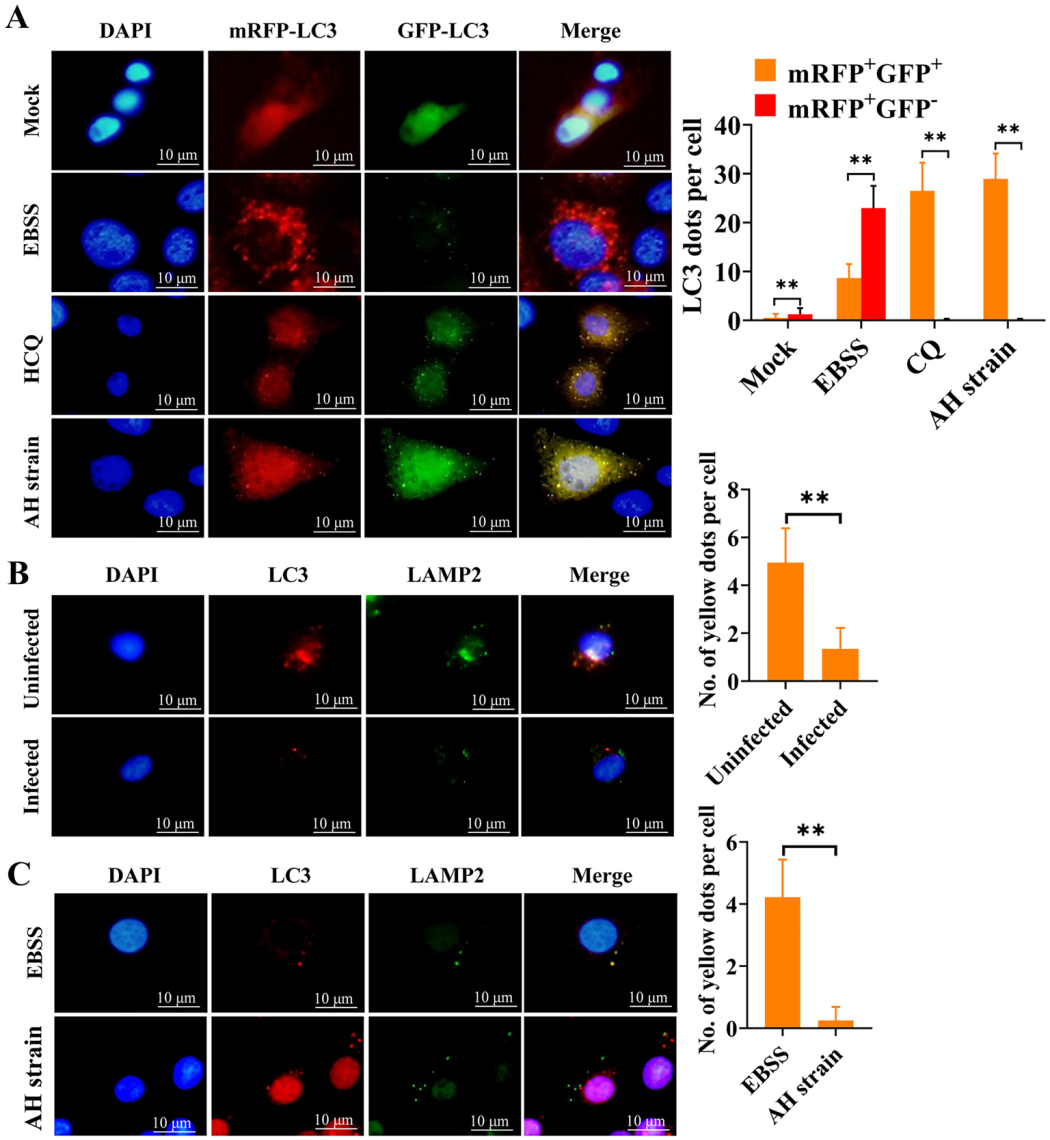
**Colocalization of autophagosomes and lysosomes analysed by immunofluorescence**. **A** Immunofluorescence of 3D4/21 cells that were infected with Ad-mRFP-GFP-LC3 and mock-infected, cultured in EBSS, treated with HCQ, or infected with AH at an MOI of 200 after 6 h. Thirty-two cells were randomly selected from each group; the yellow and red dots were counted for each cell. **B** Immunofluorescence of uninfected and infected PAMs collected from pigs and cultured in 24-well plates for 9 h. Yellow fluorescent puncta indicate the overlap of Mhp366 (green) and LAMP2 (red). Twenty cells were randomly selected from each group, and the yellow fluorescent puncta were counted from each cell. **C** Immunofluorescence of 3D4/21 cells cultured in EBSS medium or infected with AH after 6 h. Forty cells were randomly selected from each group, and the yellow fluorescent puncta were counted for each cell. Between-group differences were assessed using a *t* test; **p ≤ 0.01.

To further characterize whether *M. hyopneumoniae* infection blocks the fusion of autophagosomes with lysosomes, we employed the adenovirus tandem reporter Ad-mRFP-GFP-LC3 for the infection of 3D4/21 cells. GFP of the tandem autophagosome reporter is sensitive and unstable in the acidic compartment of lysosomes, whereas mRFP still emits red fluorescence in low-pH surroundings. Therefore, the fusion of autophagosomes and lysosomes results in the loss of green fluorescence, causing the change of fluorescence from yellow to red, making it possible to distinguish between autophagosomes (yellow) and autolysosomes (red). In mock-infected cells, fewer yellow autophagic vacuoles were visible compared to mRFP-positive autolysosomes (*p* ≤ 0.01). With the induction of complete autophagy, in EBSS-starved cells, a large amount of red fluorescence was acquired (*p* ≤ 0.01). However, large amounts of yellow fluorescent puncta formed by the superposition of red and green signals appeared in AH-infected cells under a fluorescence microscope (*p* ≤ 0.01), similar to HCQ-treated cells, where the fusion of autophagosomes and lysosomes was suppressed (*p* ≤ 0.01) (Figure 5A). Moreover, we tracked the lysosomes and autophagosomes using LAMP2 and LC3, respectively. LAMP2 did not overlap with LC3 in freshly collected *M. hyopneumoniae*-positive PAMs (Figure 5B) and AH-infected 3D4/21 cells (Figure 5C). In contrast, more yellow fluorescent puncta that were formed by the colocalization of red LC3 and green LAMP2 were observed in *M. hyopneumoniae*-free PAMs (Figure 5B) and 3D4/21 cells cultured in EBSS medium (Figure 5C). These results indicated that *M. hyopneumoniae* infection induced incomplete autophagy by blocking autophagosome-lysosome fusion.

### Incomplete autophagy promotes the intracellular proliferation of *M. hyopneumoniae* in porcine alveolar macrophages

**Figure 6:**
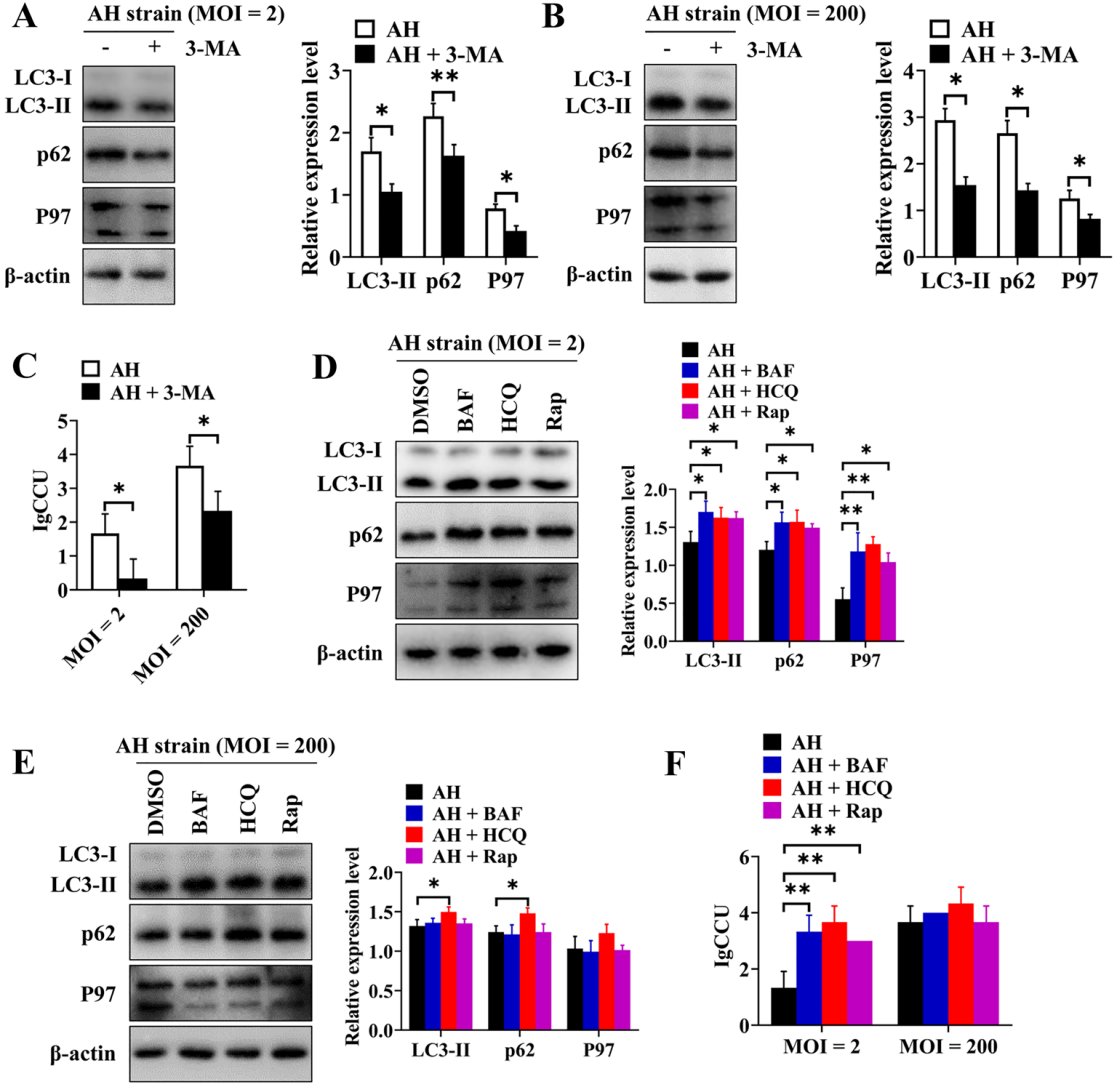
**Incomplete autophagy promotes the proliferation of** ***M. hyopneumoniae*** **in 3D4/21 cells**. **A** Western blotting of LC3-II, p62, and P97 after 3D4/21 cells were pretreated with 3-MA and then infected with AH at an MOI of 2. **B** Western blotting of LC3-II, p62, and P97 after 3D4/21 cells were pretreated with 3-MA and then infected with AH at an MOI of 200. **C** Effect of 3-MA on the reproduction of AH in 3D4/21 cells at different MOIs. **D** Western blotting of LC3-II, p62, and P97 after infecting 3D4/21 cells with AH at an MOI of 2 and treating them with different autophagy regulators for 6 h. **E** Western blotting of LC3-II, p62, and P97 after infecting 3D4/21 cells with AH at an MOI of 200 and treating them with different autophagy regulators for 6 h. **F** Effects of different autophagy regulators on the proliferation of intracellular AH in 3D4/21 cells at an MOI of 2 and an MOI of 200. Mean ± SD values from 3 independent experiments were compared using a *t* test (**A**, **B**, **C**) or one-way ANOVA (D, E, F); *p ≤ 0.05, **p ≤ 0.01.

We used several autophagy regulators to analyse the effect of autophagy on the proliferation of *M. hyopneumoniae* in PAMs. First, we determined the effects of treatment of 3D4/21 cells with 3-MA on the expression of P97 protein and the intracellular proliferation of *M. hyopneumoniae*. Compared to the mock-treated group, the expression levels of LC3-II and p62 decreased significantly in 3-MA-treated 3D4/21 cells that were infected with AH at an MOI of 2 (LC3-II, *p* ≤ 0.05; p62, *p* ≤ 0.01) (Figure 6A) or an MOI of 200 (*p* ≤ 0.05) (Figure 6B). These results proved that 3-MA inhibited the formation of autophagosomes. Moreover, in the 3-MA-treated group, the expression of P97 (*p* ≤ 0.05) (Figures 6A and B) and the CCU of the AH strain in cells also showed significant decreases (*p* ≤ 0.05) (Figure 6C), and these effects were unrelated to the MOI. These findings showed that inhibition of autophagy was not conducive to the proliferation of *M. hyopneumoniae* within PAMs.

Next, we used rapamycin to induce the production of autophagosomes and HCQ or BAF to allow the accumulation of autophagosomes in the basic autophagy process. After infection with the AH strain at an MOI of 2, cells treated with Rap/HCQ/BAF showed increased expression levels of LC3-II and p62 (Figure 6D), indicating that AH induced incomplete autophagic flux in these cells. Compared with the control group, AH-infected cells treated with Rap/HCQ/BAF had significantly greater expression of P97 (Figure 6D), indicating that autophagy increased the expression of the P97 protein (due to Rap) and prevented AH from being degraded by hydrolases in the lysosomes (due to HCQ/BAF). After infection of 3D4/21 cells with AH at an MOI of 200, there was no significant change in the expression of LC3-II and p62 in cells treated with Rap/BAF (*p* > 0.05), indicating that infection of 3D4/21 cells with AH at a high MOI saturated autophagosome formation, although the expression levels of the LC3-II and p62 proteins in the HCQ treatment group increased significantly (*p* ≤ 0.05) (Figure 6E). Moreover, the expression of P97 protein did not change significantly (*p* > 0.05) in AH-infected cells treated with Rap/HCQ/BAF at an MOI of 200 (Figure 6E). When the cells were infected with the AH strain at an MOI of 2, compared with the control group, the CCU of the AH strain in the Rap/HCQ/BAF treatment group was significantly higher (*p* ≤ 0.01) (Figure 6F). However, when the cells were infected with AH at an MOI of 200, there was no significant difference in the CCU of AH in the Rap/HCQ/BAF-treated group compared with the control group (*p* > 0.05) (Figure 6F). Therefore, the above results demonstrated that incomplete autophagy promoted the proliferation of *M. hyopneumoniae* and that a high MOI of *M. hyopneumoniae* saturated the formation of autophagosomes in cells.

### *M. hyopneumoniae* induces autophagy via the JNK and Akt signalling pathways

**Figure 7:**
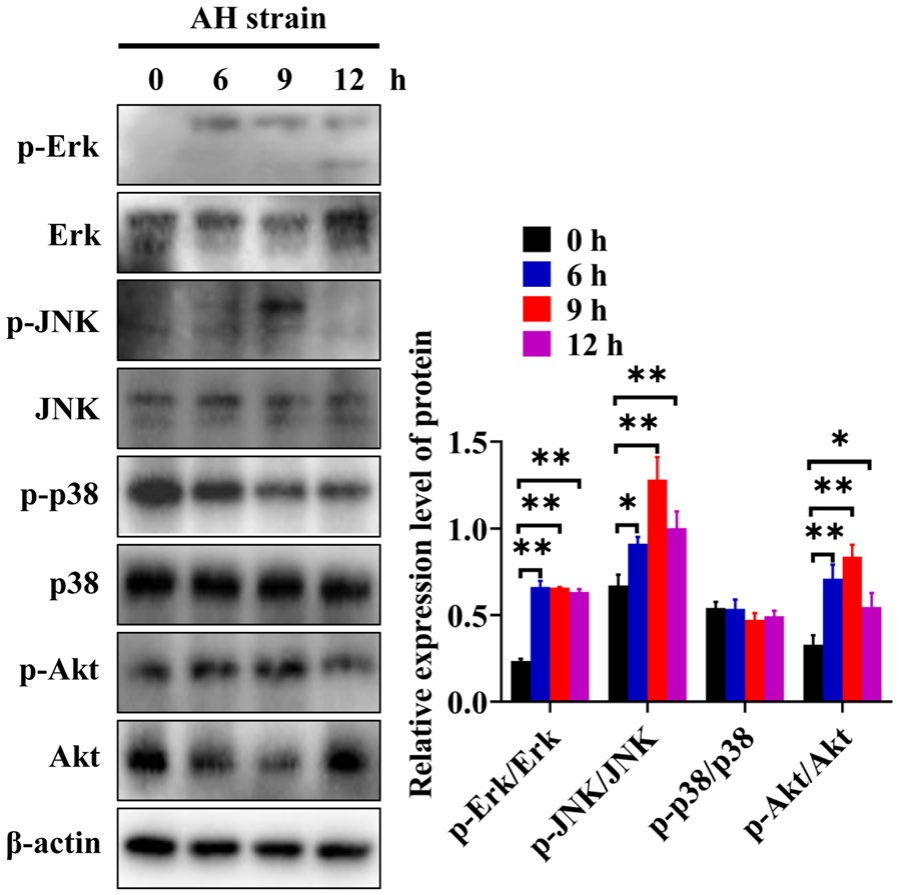
**AH infection of 3D4/21 cells activates multiple signalling pathways. Western blotting of the phosphorylation of Erk1/2, JNK, p38, and Akt at different time points after infection of 3D4/21 cells by AH**. Mean ± SD values from 3 independent experiments are presented and compared using one-way ANOVA. *p ≤ 0.05, **p ≤ 0.01.

Evidences have shown that MAPK, such as Erk1/2, JNK and p38 MAPK, and Akt signalling pathways are involved in various aspects of cellular physiology, including inflammation, apoptosis, and autophagy [15–18]. Several pathogens participate in autophagy through the MAPK or Akt signalling pathways [17, 19–22]. To preliminarily explore the relationship between autophagy induced by *M. hyopneumoniae* and the corresponding signalling pathways, we evaluated the phosphorylation of Erk1/2, JNK, p38, and Akt in AH-infected 3D4/21 cells at different time points. AH-infected 3D4/21 cells showed induction of autophagy based on the increased expression of LC3-II and Beclin 1 (Additional file 4). Similarly, the phosphorylation of Erk1/2, JNK and Akt was significantly promoted after autophagy induction in 3D4/21 cells (Figure 7). Notably, the timing of JNK and Akt phosphorylation was in parallel with the increased expression of LC3-II and Beclin 1, with maximal expression observed at 9 h after AH infection. Nevertheless, there was no significant difference between the infected and control groups with respect to the phosphorylation of p38.

**Figure 8:**
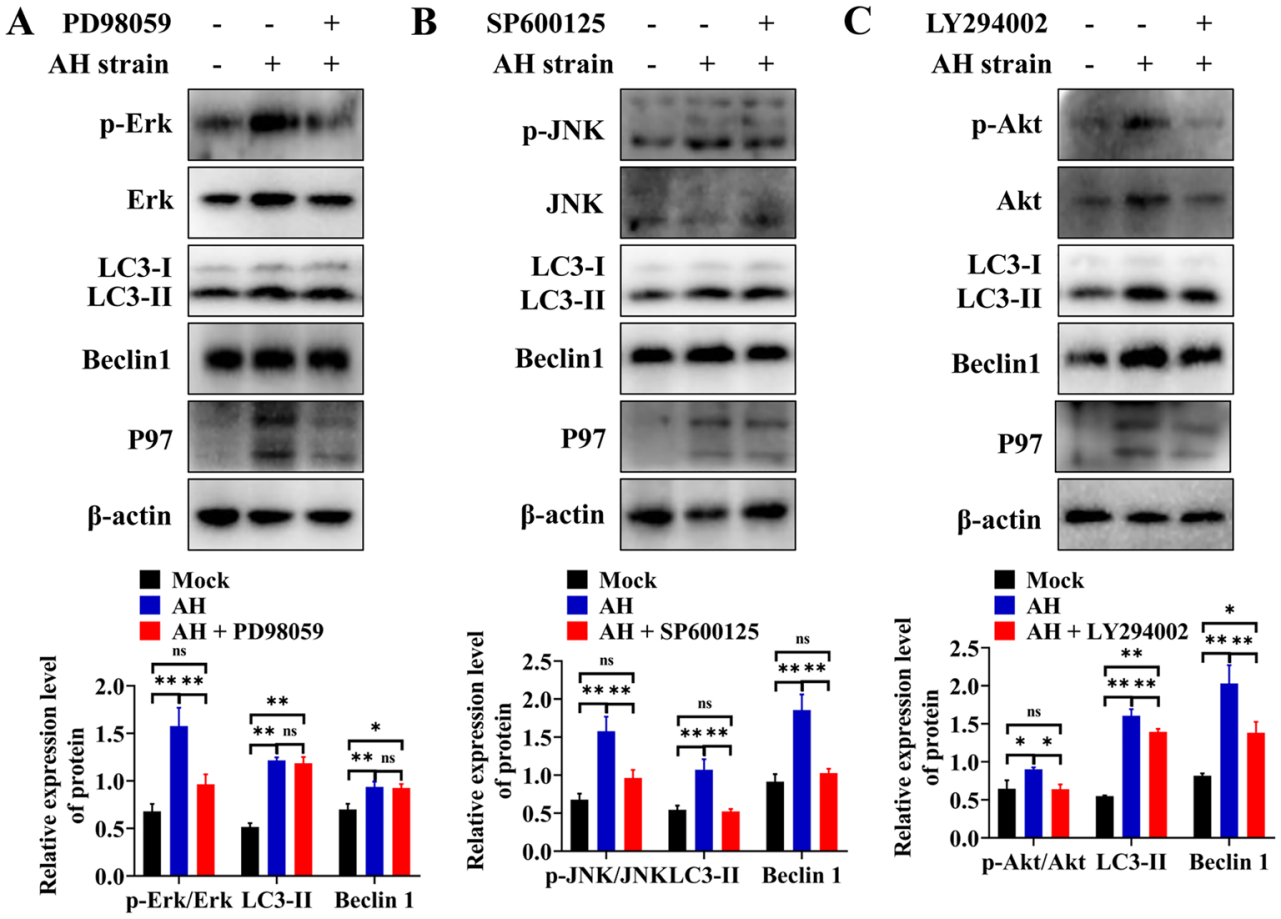
**Blocking the JNK and Akt pathways decreases AH-induced autophagy in 3D4/21 cells**. **A** Effect of an Erk1/2 inhibitor (PD98059) on the phosphorylation of Erk1/2 and the expression of LC3-II and Beclin 1. **B** Effect of a JNK inhibitor (SP600125) on JNK phosphorylation and LC3-II and Beclin 1 expression. **C** Effect of an Akt signalling pathway inhibitor (LY294002) on Akt phosphorylation and LC3-II and Beclin 1 expression. Mean ± SD values from 3 independent experiments are presented and compared using one-way ANOVA; ns: not significant, *p ≤ 0.05, **p ≤ 0.01.

**Figure 9:**
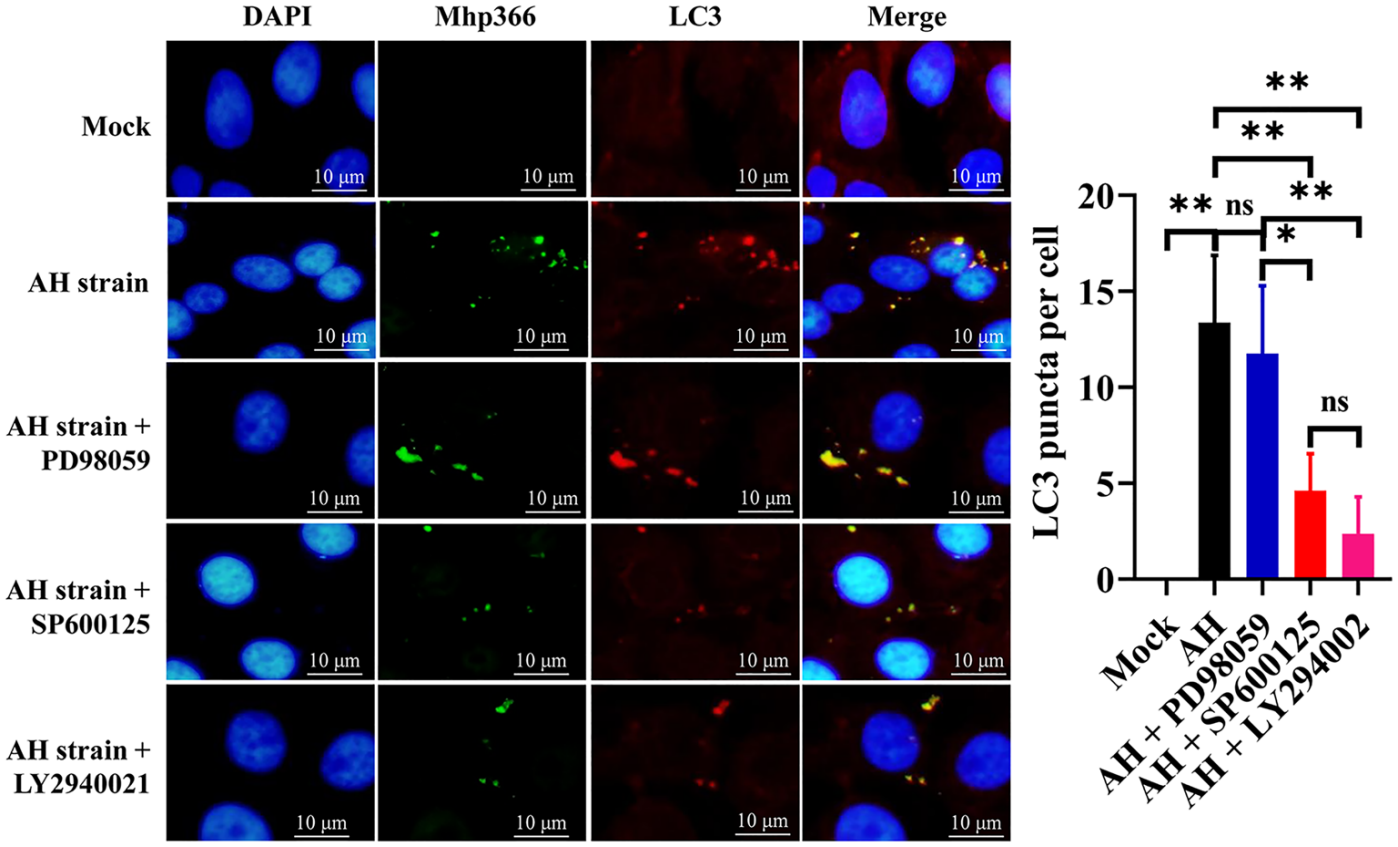
**Changes in LC3 fluorescent puncta in AH-infected 3D4/21 cells after treatment with different autophagy inhibitors**. Mean ± SD values from 8 independent experiments were compared using one-way ANOVA; ns: not significant, *p ≤ 0.05, **p ≤ 0.01.

To explore whether the increase in phosphorylation of these proteins was related to autophagy, 3D4/21 cells were pretreated with PD98059, SP600125, and LY294002, the specific inhibitors of Erk1/2, JNK and Akt, respectively, for 1 h before infection by AH, and then the expression levels of autophagy-related proteins and the phosphorylation of Erk1/2, JNK and Akt were evaluated. The results showed significant decreases in the phosphorylation of Erk1/2, JNK, and Akt after treatment with the corresponding inhibitor (Figure 8), indicating that these inhibitors inhibited the phosphorylation of the corresponding proteins. The expression levels of LC3-II and Beclin1 remained unchanged (*p* > 0.05) (Figure 8A) in AH-infected 3D4/21 cells pretreated with PD98059, demonstrating that the Erk1/2 signalling pathway had no effect on AH-induced autophagy in 3D4/21 cells. However, the expression levels of LC3-II and Beclin 1 were significantly decreased after pretreatment of AH-infected cells with SP600125 (*p* ≤ 0.01) (Figures 8B) or LY294002 (*p* ≤ 0.01) (Figure 8C). Immunofluorescence analysis of 3D4/21 cells indicated that the number of fluorescent puncta of LC3 decreased significantly after treatment with SP600125 (*p* ≤ 0.01) and LY294002 (*p* ≤ 0.01), but PD98059 had no significant effect (*p* > 0.05) (Figure 9). These results indicated the involvement of the JNK and Akt signalling pathways in *M. hyopneumoniae*-induced autophagy in PAMs.

## Discussion

Infection of pigs by the respiratory pathogen *M. hyopneumoniae* is a significant problem for the pig industry. However, the pathogenetic mechanisms of this infection are not well characterized. One study showed that *M. hyopneumoniae* can invade porcine type I and type II alveolar cells and PAMs [9], and another study showed that *M. hyopneumoniae* can induce autophagy in lung cells [10]. However, no previous study has identified the specific types of lung cells in which autophagy was induced by this pathogen. PAMs play important roles in innate and adaptive immune responses induced by respiratory pathogens [23, 24]. In the present study, we examined whether *M. hyopneumoniae* can induce autophagy—a type of innate immune response—after entering PAMs.

To the best of our knowledge, this is the first study to demonstrate that *M. hyopneumoniae* infection leads to the accumulation of autophagosomes in PAMs by triggering autophagy through the JNK and Akt signalling pathways. We also showed that autophagy promoted the proliferation of *M. hyopneumoniae* in a manner that blocked the fusion of autophagosomes and lysosomes, which is a mechanism for the digestion of autophagosome contents by lysosomal enzymes in a low-pH microenvironment.

As an innate immune response mechanism, autophagy has been shown to be involved in the elimination of some intracellular pathogens, such as *Salmonella* Typhimurium [25], *Mycobacterium tuberculosis* [26], and *Aggregatibacter actinomycetemcomitans* [27]. However, studies have shown that some pathogens have evolved molecular strategies to manipulate the autophagic process for their own benefit. For example, the NP and M2 proteins of influenza A virus were shown to induce autophagy, which in turn promoted viral replication [28]. A complete autophagic response was shown to benefit the classical swine fever virus yield, as depletion of endogenous Beclin 1 or LC3 downregulated the production of the virus [29]. Some pathogens can block the fusion of autophagosomes and lysosomes after their entry into cells, which leads to the accumulation of autophagosomes; this blocks the recycling of LC3 and prevents the degradation of p62 in autolysosomes. For example, human parainfluenza virus type 3 infection was shown to induce incomplete autophagy by promoting the accumulation of autophagosomes that do not fuse with lysosomes [30]. Another study demonstrated the accumulation of autophagosomes and a reduction in autophagic flux after infection of the human myeloid cell line PLB-985 with adherent-invasive *Escherichia coli* [31]. We studied freshly harvested PAMs and cultured CD4/21 cells using TEM to detect autophagosomes; in addition, we performed Western blotting to measure autophagic marker proteins and immunofluorescence to determine the presence of LC3 puncta. The results showed that *M. hyopneumoniae* entered PAMs and induced the formation of autophagosomes, followed by blockade of the fusion of autophagosomes and lysosomes, based on the levels of LC3-II and p62, colocalization of autophagosomal and lysosomal proteins, and other key cellular events. The above results confirmed that *M. hyopneumoniae* infection blocked autophagic flux. Our results were similar to some cellular events caused by infection with other *Mycoplasma* species. For example, *M. bovis* infection was shown to induce autophagy but subvert the maturation of autophagosomes, leading to blockade of autophagic flux [32]. *M. pulmonis* infection was shown to cause the accumulation of p62, a cargo adaptor for autophagic degradation [33]. Our results indicated that autophagosomes encapsulated *M. hyopneumoniae*, resulting in the inability to fuse autophagosomes and lysosomes, which may be an important reason for the persistent infection of the host respiratory system caused by *M. hyopneumoniae* [34].

Several studies have shown that incomplete autophagy not only facilitates the accumulation of autophagosomes but also promotes pathogen proliferation. Coxsackievirus A16 infection has been shown to trigger the generation of autophagosomes by impairing autophagosome maturation, resulting in increased extracellular virus production [21]. The available data indicate that a number of bacterial species also achieve survival and proliferation in cells by blocking autophagic flux, such as *S. aureus* [35] and adherent-invasive *E. coli* [31]. *M. hyopneumoniae* may not be killed directly or presented to the cell surface after antigen processing by PAMs due to incomplete induction of autophagy, thus allowing its colonization and proliferation within macrophages. To understand the influence of incomplete autophagy on the survival of *M. hyopneumoniae*, we measured the CCU of *M. hyopneumoniae* after treating 3D4/21 cells with different autophagy regulators. The results showed that blocking the initiation of autophagy and the formation of autophagosomes reduced the survival of *M. hyopneumoniae* in PAMs; however, inducing autophagosome formation or blocking the fusion of autophagosomes and lysosomes promoted the reproduction of *M. hyopneumoniae* in PAMs. This suggested that preventing acidification by lysosomes is an important reason for the survival of *M. hyopneumoniae*. Previous research indicates that the *A. baumannii* OmpA protein disrupts the maturation of autophagosomes in HeLa cells and interferes with the fusion of lysosomes, thus triggering incomplete autophagy [6]. Future research should examine the specific proteins in *M. hyopneumoniae* that block the fusion of autophagosomes with lysosomes.

Several studies conducted in the last decade have shown that pathogens can regulate autophagy via different signalling pathways. For instance, Coxsackievirus A16 was shown to induce autophagosome formation by inhibiting Akt/mTOR signalling and activating Erk signalling [21]. Seneca Valley virus was shown to modulate autophagy via the p38, Erk1/2 and Akt pathways [36]. Some studies have examined the signalling pathway of autophagy induced by other porcine respiratory pathogenic bacteria. For example, in an in vitro study, *Glaesserella parasuis* (*Haemophilus parasuis*) was found to induce autophagy in 3D4/21 cells via the AMPK signalling pathway [37]. Some *Mycoplasma* species induce autophagy via the MAPK signalling pathway. For instance, inhibition of Erk1/2 phosphorylation significantly attenuated the level of autophagy after *M. gallisepticum* infection [19]. Luo et al. [38] showed that activation of the NOD2 and JNK pathways is required for autophagy in *M. ovipneumoniae*-infected cells. In this study, we examined the signalling pathway of autophagy induced by *M. hyopneumoniae*, and the results indicated that *M. hyopneumoniae* induced autophagy via the JNK and Akt signalling pathways, although autophagy induced by veterinary pathogenic bacteria via the Akt pathway is rarely reported [39, 40].

In summary, this study demonstrated for the first time that *M. hyopneumoniae* induced incomplete autophagy in freshly collected PAMs and in an established line of cultured PAMs, resulting in enhanced replication of *M. hyopneumoniae* in host cells. The process of incomplete autophagy occurs via activation of the JNK and Akt signalling pathways. These results provide important insights into the pathogenesis of *M. hyopneumoniae* infection.

## Supplementary Information

**Additional file 1**. **Flow diagrams of autophagic flux measurement, analysis of signalling pathways, and detection of the influence of autophagy on the intracellular proliferation of M. hyopneumoniae**. (A) Detection of autophagic flux; (B) identification of signalling pathways that initiate autophagy induced by M. hyopneumoniae; (C) analysis of the effect of autophagy on the proliferation of M. hyopneumoniae in 3D4/21 cells.

**Additional file 2**. **Effects of different concentrations of the four autophagy regulators on the expression of LC3-II and p62 in 3D4/21 cells**. (A) 3-MA; (B) rapamycin; (C) HCQ; (D) BAF. Mean ± SD values from 3 independent experiments were compared using one-way ANOVA. *p ≤ 0.05, **p ≤ 0.01.

**Additional file 3**. **Cytotoxic effects of four autophagy regulators on 3D4/21 cells at their optimal working concentrations**. 3-MA: 5 mmol/L; rapamycin: 1000 nmol/L; HCQ: 25 µmol/L; BAF: 200 nmol/L. Mean ± SD values from 3 independent experiments were compared using one-way ANOVA. ns: not significant.

**Additional file 4**. **Western blotting of autophagy-related proteins (LC3-II and Beclin 1) at different time points after infection of 3D4/21 cells by AH**. Mean ± SD values from 3 independent experiments were compared using one-way ANOVA; *p ≤ 0.05, **p ≤ 0.01.

## Data Availability

The datasets supporting the conclusions of this article are included within the article text and additional files.
