# Supplementary figures and images for: Incomplete autophagy promotes the proliferation of Mycoplasma hyopneumoniae through the JNK and Akt pathways in porcine alveolar macrophages
Source: Vet Res. 2022 Aug 4;53:62. doi: 10.1186/s13567-022-01074-5 (PMC9351181; doi:10.1186/s13567-022-01074-5)

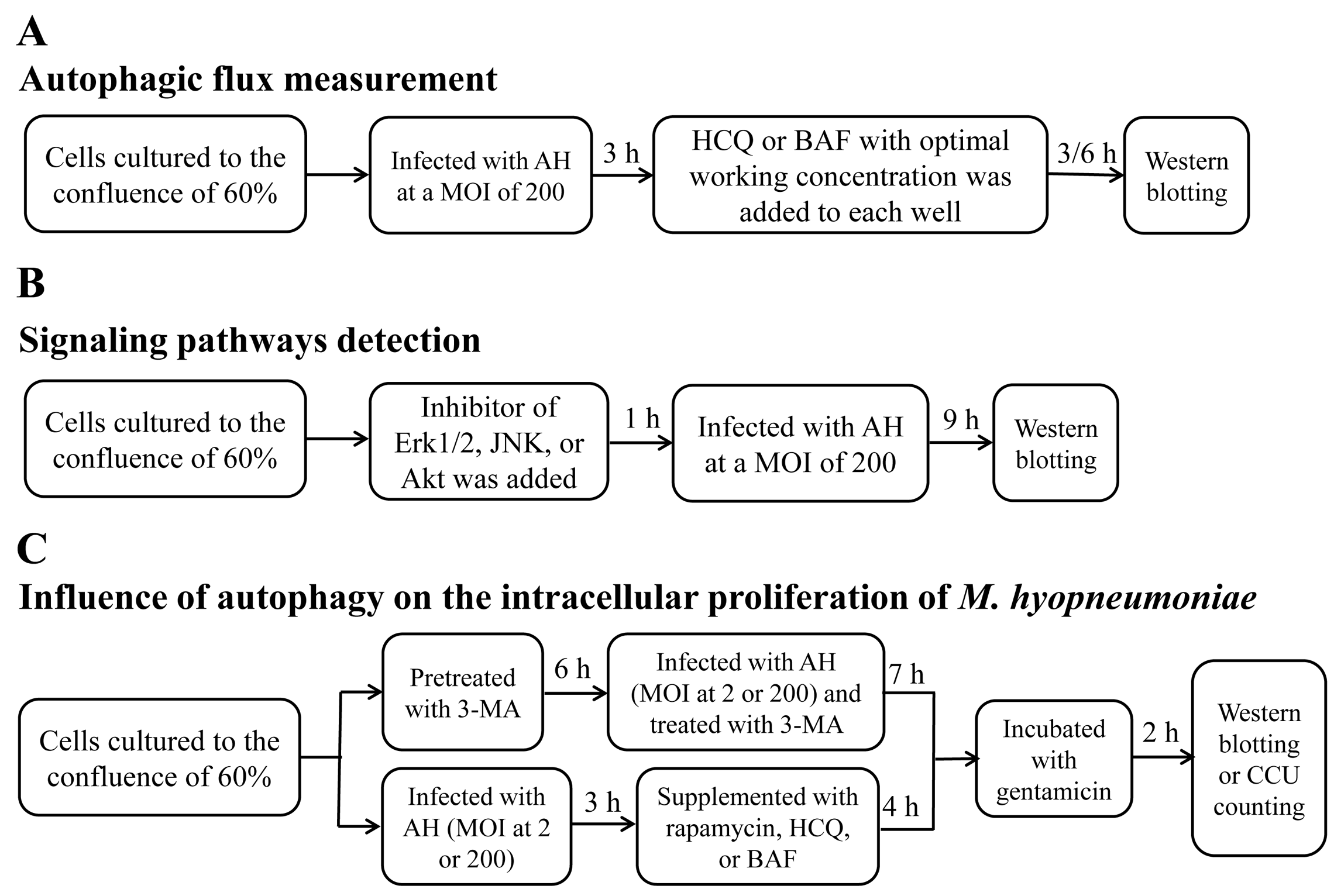

Supplement: Supplementary file 1 — Additional file 1. Flow diagrams of autophagic flux measurement, analysis of signalling pathways, and detection of the influence of autophagy on the intracellular proliferation of M. hyopneumoniae. (A) Detection of autophagic flux; (B) identification of signalling pathways that initiate autophagy induced by M. hyopneumoniae; (C) analysis of the effect of autophagy on the proliferation of M. hyopneumoniae in 3D4/21 cells. [file 13567_2022_1074_MOESM1_ESM.tif]

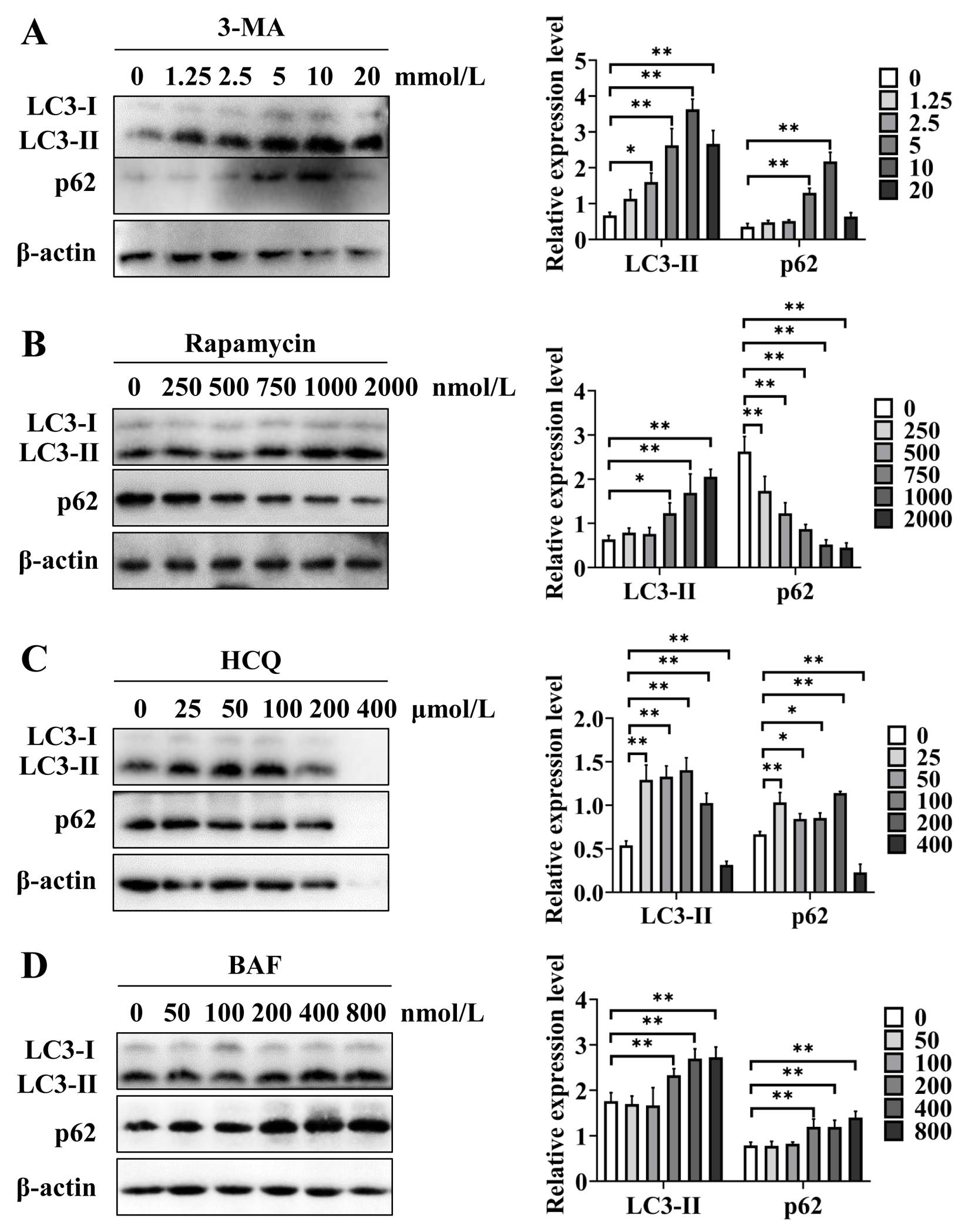

Supplement: Supplementary file 2 — Additional file 2. Effects of different concentrations of the four autophagy regulators on the expression of LC3-II and p62 in 3D4/21 cells. (A) 3-MA; (B) rapamycin; (C) HCQ; (D) BAF. Mean ± SD values from 3 independent experiments were compared using one-way ANOVA. *p ≤ 0.05, **p ≤ 0.01. [file 13567_2022_1074_MOESM2_ESM.tif]

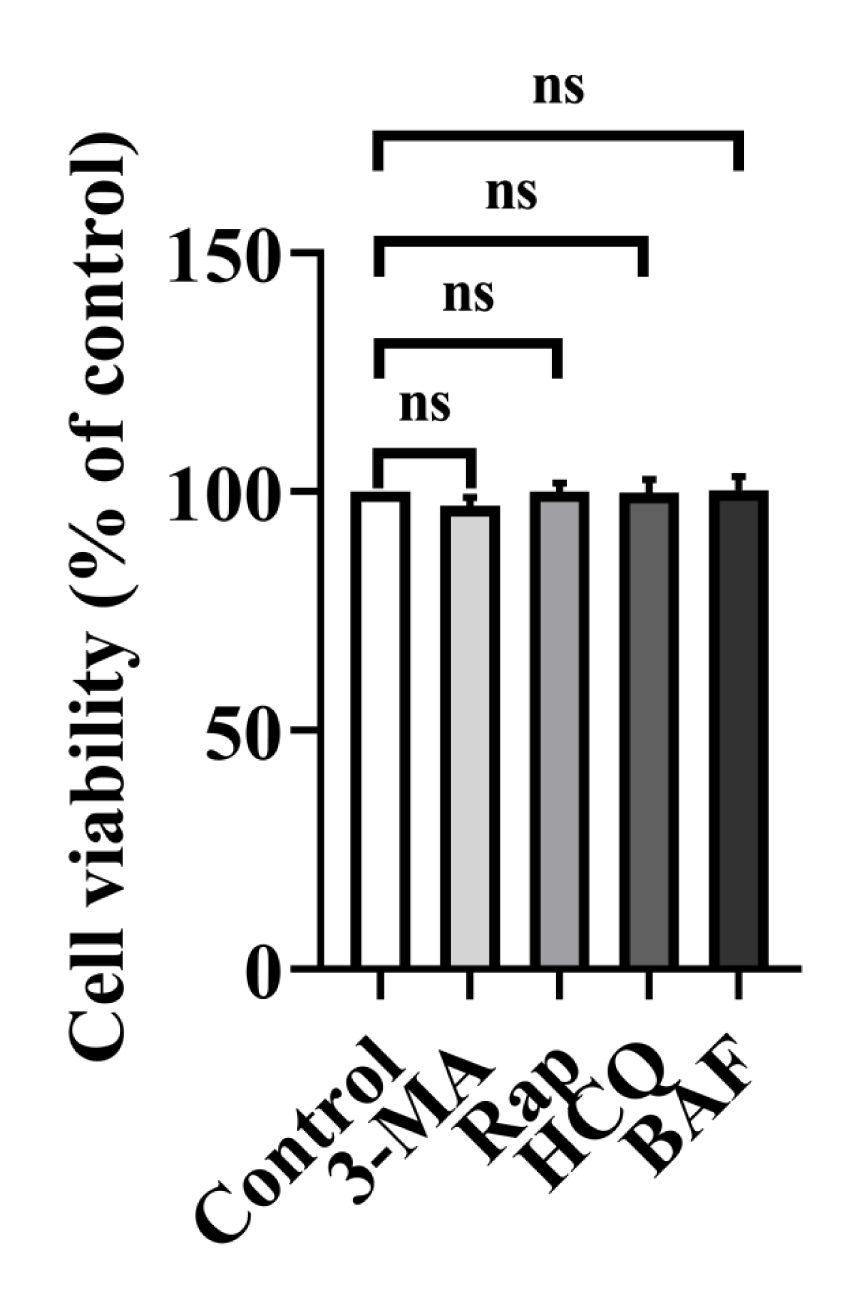

Supplement: Supplementary file 3 — Additional file 3. Cytotoxic effects of four autophagy regulators on 3D4/21 cells at their optimal working concentrations. 3-MA: 5 mmol/L; rapamycin: 1000 nmol/L; HCQ: 25 µmol/L; BAF: 200 nmol/L. Mean ± SD values from 3 independent experiments were compared using one-way ANOVA. ns: not significant. [file 13567_2022_1074_MOESM3_ESM.tif]

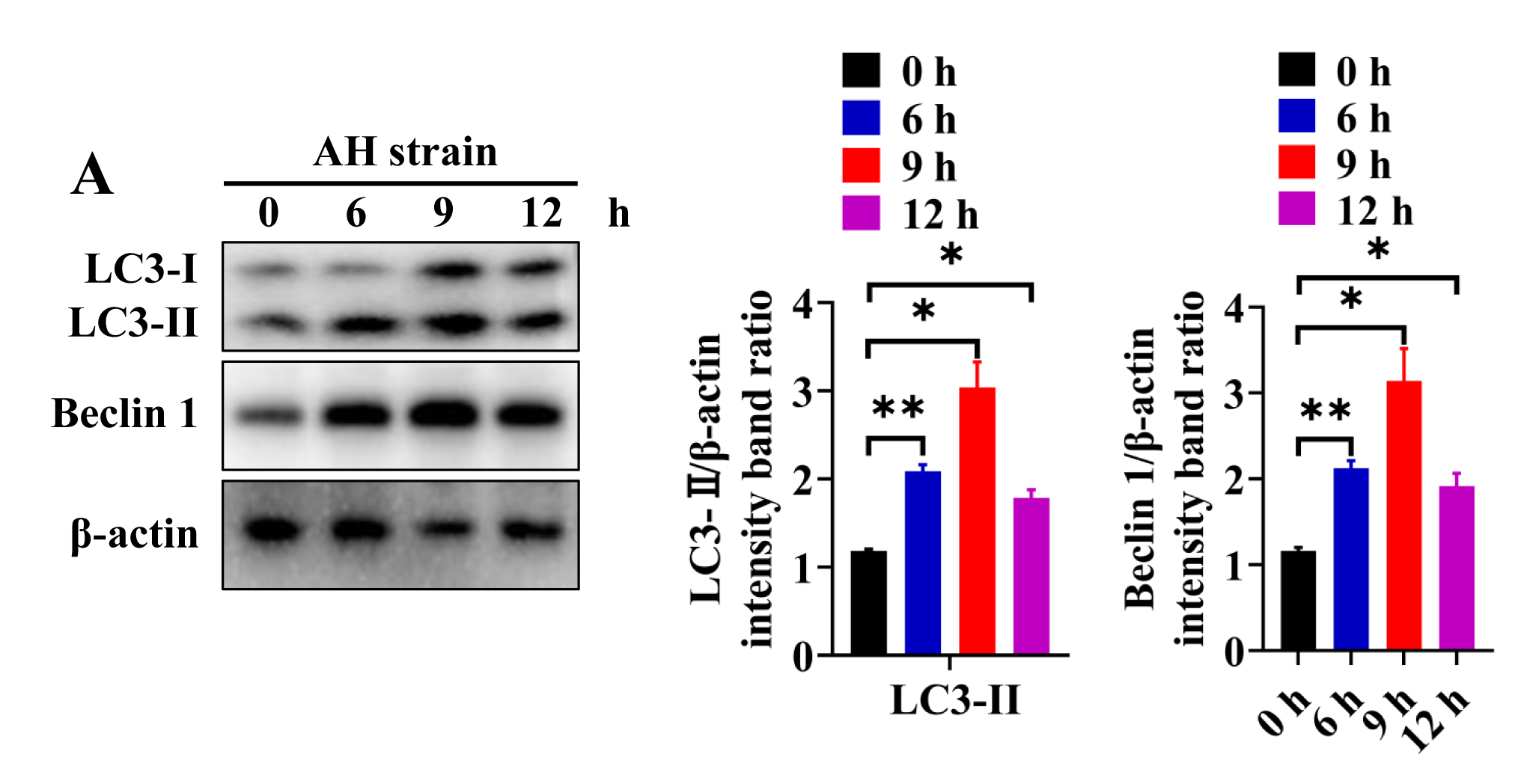

Supplement: Supplementary file 4 — Additional file 4. Western blotting of autophagy-related proteins (LC3-II and Beclin 1) at different time points after infection of 3D4/21 cells by AH. Mean ± SD values from 3 independent experiments were compared using one-way ANOVA; *p ≤ 0.05, **p ≤ 0.01. [file 13567_2022_1074_MOESM4_ESM.tif]
